# Supplementary material for: AML patient blasts exhibit polarization defects upon interaction with bone marrow stromal cells
Source: EMBO Rep. 2025 Jun 11;26(13):3264–79. doi: 10.1038/s44319-025-00466-w (PMC12238381; doi:10.1038/s44319-025-00466-w)
Supplement: Supplementary file 2 — Movie EV1 [file 44319_2025_466_MOESM2_ESM.zip › Movie EV1.rtf]

Movie EV1: Leukemic cells originating from AML cell lines lack the motility observed in AML patient blasts within a healthy mesenchymal stromal cell compartment in the BMoC model.AML cell line (MOLM-14, left) and AML patient blasts (right) interacting with MSC collected from healthy donor were recorded within the BMoC. Live-imaging was performed  in transmitted light with a 20X magnifying objective. Images were acquired every two-minute over a period of 22 hours. The video is displayed at a framerate of 7 frames per second.
