# Supplementary material for: AML patient blasts exhibit polarization defects upon interaction with bone marrow stromal cells
Source: EMBO Rep. 2025 Jun 11;26(13):3264–79. doi: 10.1038/s44319-025-00466-w (PMC12238381; doi:10.1038/s44319-025-00466-w)
Supplement: Supplementary file 3 — Expanded View Figures [file 44319_2025_466_MOESM3_ESM.pdf]

Expanded View Figures

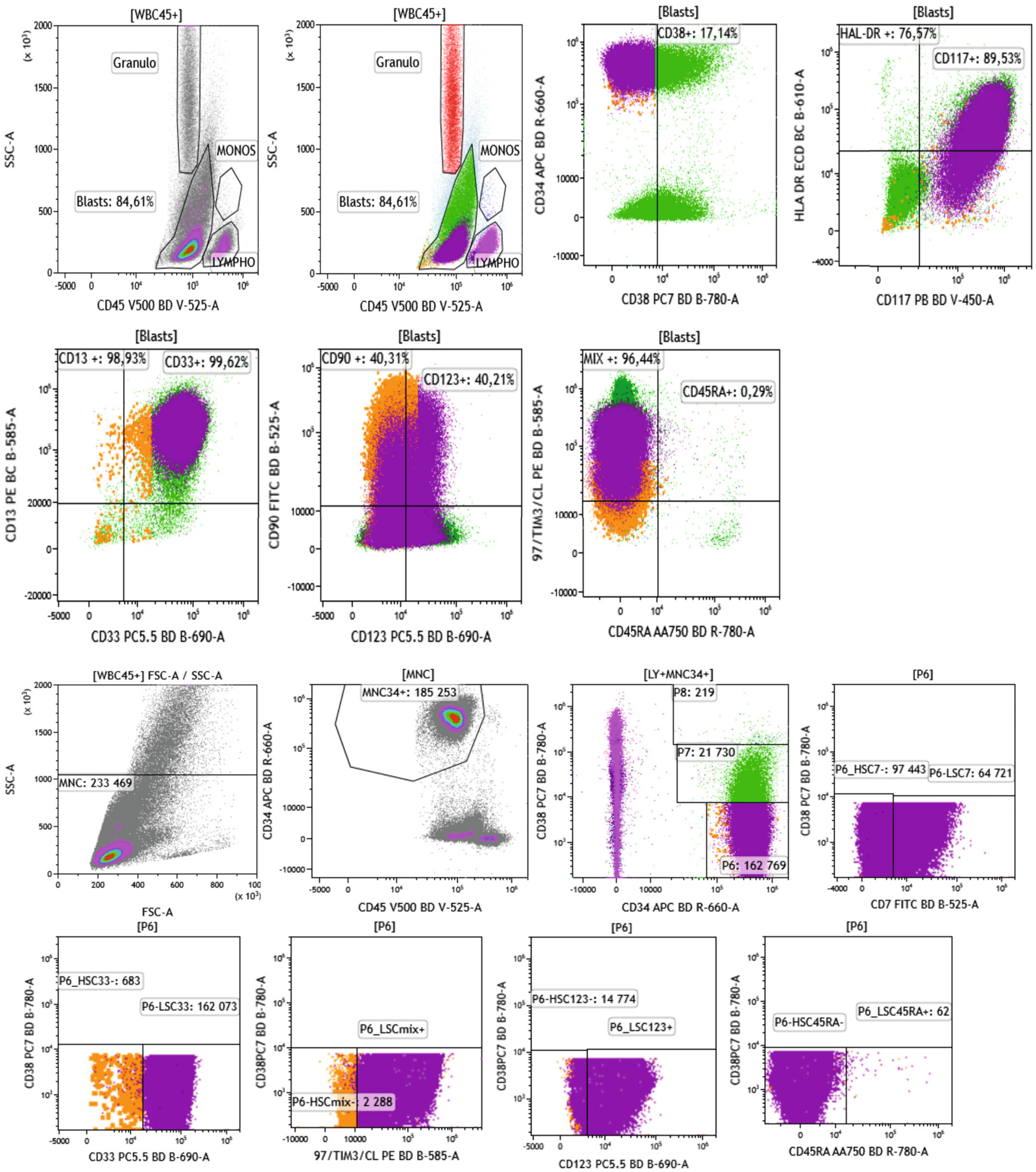

Figure EV1. AML patient n° 10 hematopoietic cell flow cytometry gating strategy (blasts and LSCs).

Flow cytometry analysis of an AML patient with a high LSC fraction.

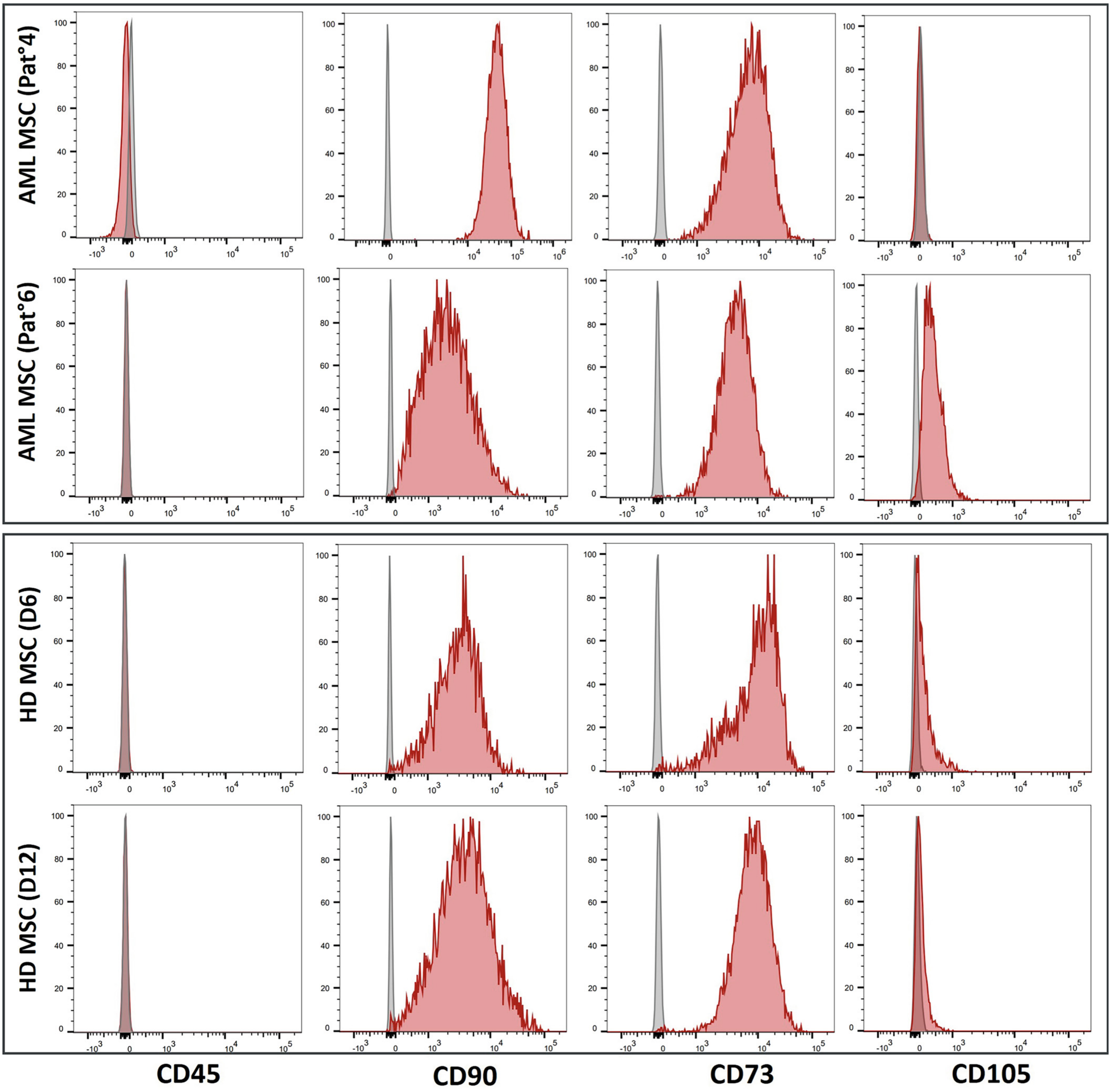

Figure EV2. AML patient immunophenotypic profiles of mesenchymal stromal cells (MSCs).

Flow cytometry strategy to characterize from the AML patient MSCs considered as CD45, CD90, CD73, CD105.

 © The Author(s)

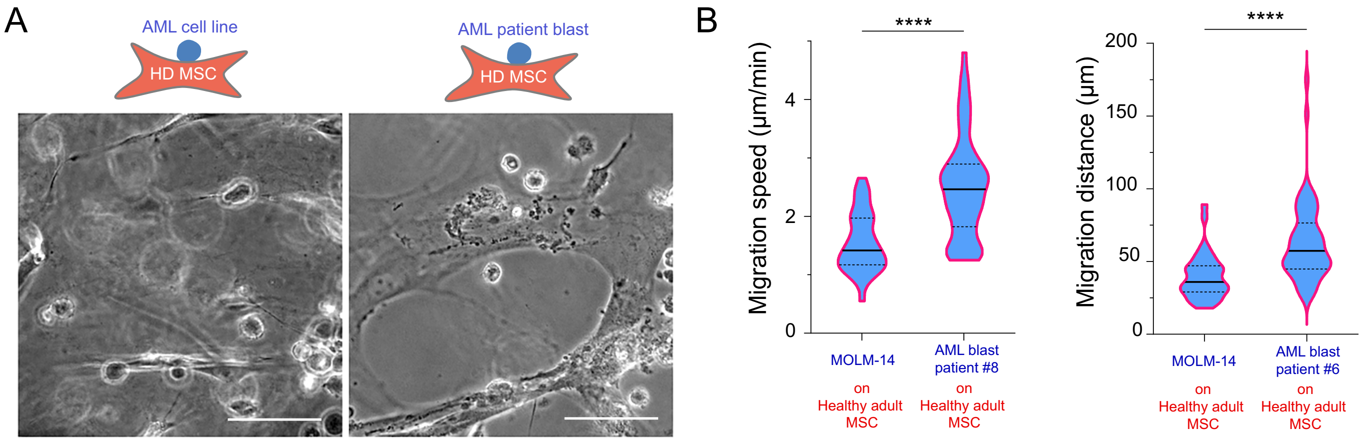

Figure EV3. Leukemic cells originating from AML cell lines lack the motility observed in AML patient blasts within a healthy mesenchymal stromal cell compartment in the BMoC model.

(**A**) AML cell line (MOLM-14) and AML patient blasts (blue in the schematics) were loaded in the BMOC and thus interacted with MSC from healthy donors (orange in the schematics). Images in transmitted light were extracted from time-lapse movies of AML cell lines and patient blast within healthy compartments. Scale bars correspond to 50 µm. (**B**) Quantification of the mean speed and traveled distance during 20 min of the migration of leukemic cells (MOLM-14) or AML patient blast in a healthy stromal compartment (HD MSC). Tracks were analyzed at various positions within the stromal compartment ($n_{\text{MOLM-14/ HD MSC}} = 41$, $n_{\text{AML blast/ HD MSC}} = 77$). In the violin plots, black bars represent the median and dashed lines the 95% confidence interval. Differences between populations were evaluated using a Mann–Whitney test with *P* values < 0.0001 (****).

© The Author(s)
